# A unique CO-like micrometeorite hosting an exotic Al-Cu-Fe-bearing assemblage – close affinities with the Khatyrka meteorite

**DOI:** 10.1038/s41598-019-48937-0

**Published:** 2019-08-27

**Authors:** M. D. Suttle, K. Twegar, J. Nava, R. Spiess, J. Spratt, F. Campanale, L. Folco

**Affiliations:** 1 Dipartimento di Scienze della Terra, Università di Pisa, 56126 Pisa, Italy; 2 Department of Chemistry, Istanbul Technological University, 34467 Istanbul, Turkey; 3 Dipartimento di Geoscienze Via Gradenigo 6, 35131 Padova, Italy; 4 Department of Earth Science, The Natural History Museum, Cromwell Rd, South Kensington, London, SW7 5BD UK; 5 Center for Nanotechnology Innovation@NEST, Istituto Italiano di Tecnologia (IIT), Piazza San Silvestro 12, 56127 Pisa, Italy

**Keywords:** Mineralogy, Geochemistry, Petrology, Early solar system

## Abstract

We report the discovery of a unique micrometeorite, containing an exotic Al-Cu-Fe alloy composed of two intermixed phases: khatyrkite (CuAl_2_) and stolperite (CuAl) and both containing minor Fe (<1.4 wt%). These phases are dendritic and rapidly co-crystallized at the binary system’s peritectic (~550 °C). The host micrometeorite is an otherwise typical S-type micro-porphyritic cosmic spherule containing relict olivine (Fo76–90, Cr_2_O_3_: 0.01–0.56 wt%, MnO: 0.03–0.32 wt% and CaO: 0.09–0.22 wt%) and a cumulate layered texture. These properties suggest the micrometeorite is derived from a carbonaceous chondrite (best matched to a CO chondrite) and entered the atmosphere a high speed (~16 kms^−1^), implying an origin from a highly eccentric orbit. This particle represents the second independent discovery of naturally occurring intermetallic Al-Cu-Fe alloys and is thus similar to the previously reported Khatyrka meteorite - a CV chondrite containing near-identical alloys and the only known natural quasicrystals. We did not observe quasicrystalline phases in this micrometeorite, likely due to the low amounts of Fe in the alloy, insufficient to stabilize quasicrystals. Our discovery confirms the existence of Al-Cu-Fe intermetallic alloys on chondritic parent bodies. These unusual phases require a currently unexplained formation process, we tentatively suggest this could represent the delivery of exotic interstellar material to the inner solar system via impact.

## Introduction

Micrometeorites are grains of cosmic dust <2 mm in diameter which originate from solar system small bodies (i.e. asteroids and comets^1^). They are primarily derived from primitive early solar system remnants and are therefore commonly fragments of fine-grained matrix or anhydrous silicate-rich chondrules^2,3^) as well as rare refractory CAIs and AOAs^4^. They are classified into fine- and coarse-grained classes or a joint composite class depending on their texture which itself is a result of how the parent body fragmented^1,2^.

Upon liberation, individual cosmic dust grains rapidly spiral into the inner solar system, moving under non-gravitational Poynting–Robertson (P-R) drag^5^. This is due to radiation pressure from the Sun exerting a force tangential to the dust grain’s orbit, thereby resulting in a progressive loss in angular momentum^6^. This delivery mechanism efficiently (<10 Ma) scavenges all mm-scale dust from the inner solar system, with removal occurring through capture by the terrestrial planets or by the Sun’s photosphere^7^. Thus, micrometeorites falling to Earth originate from a large and diverse population of solar system small bodies – all those which are actively producing dust. This is in contrast to meteorites, which instead derive principally from the Kirkwood Gaps in the asteroid belt and are delivered by orbital and secular resonances^8^. Consequently, the study of micrometeorites provides a wide range of extraterrestrial material; much of which is directly related to existing meteorite groups^3,9^ but which also contains new and exotic materials that would otherwise remain unsampled and unstudied.

Examples of unique micrometeorites with unusual petrology include: a basaltic micrometeorite from an differentiated protoplanetary crust, but not originating from either Vesta, the Moon or Mars^10^, a microchondrule-bearing micrometeorite (potentially) derived from a cometary parent body^11^, refractory polycrystalline particles^12,13^ and a population of ^16^O-poor cosmic spherules possibly representing a new class of ordinary chondrites^14^. Similarly, in this study we describe a new, unique micrometeorite, showing close geochemical and mineralogical affinities to the Khatyrka meteorite^15^, owing to the presence of a reduced and highly exotic assemblage of Al-Cu-Fe alloys embedded within an otherwise typical cosmic spherule host. This particle represents the second independent discovery of naturally occurring Al-Cu-Fe alloys, whilst also representing a new petrographic setting and simultaneously providing new insights into the formation of these phases in space during the early solar system.

## Methods and Materials

**Figure 1:**
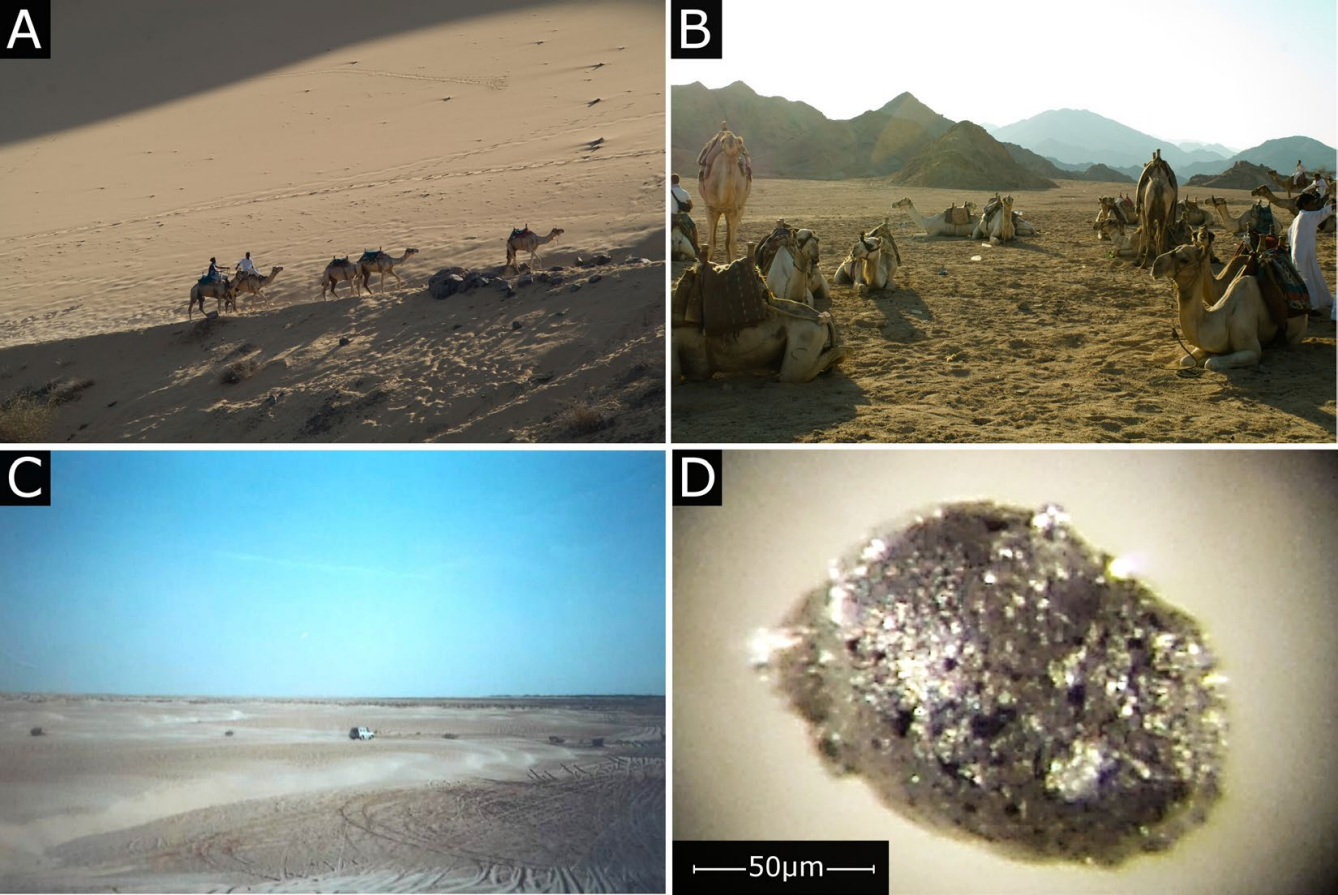
The collection of particle KT01. Images from the Nubian desert and under the optical microscope (particle external surface).

We investigate a single micrometeorite: KT01, recovered from the Nubian desert (Sudan) during a 2013 meteorite hunting expedition organized by the Amateur Meteorite Association of Edfu, Egypt. This expedition set out to recover new fragments of the Almahata Sitta meteorite; a urelite that fell on 7^th^ October 2008 (at 01:49 UTC), breaking apart at 35.7 km altitude and resulting in the generation of a strewn field up to 100 km in length^16^. During the expedition, a base camp was established at the coordinates: 20°47′35.390″N, 31°23′44.498″W, approximately 70 km west of the city of Abri. Here, in addition to visually searching the desert surface for large meteorites (Fig. 1A–C), team members investigated the desert sand under binocular microscope, looking for possible meteorite fragments and cosmic dust. Approximately 150 kg of sand were searched resulting in the discovery of 22 micrometeorites – one of which is the subject of this study (Fig. 1D). The remaining particles were found to be typical cosmic spherules, relatively common among micrometeorite collections and containing no unusual or undescribed phases.

Initial geochemical analysis on KT01 was conducted at the University of Pisa’s Interdepartmental centre for science and engineering (CISIM). The particle was analysed using backscatter electron (BSE) imaging, standard-less energy dispersive X-ray (EDX) spectrometry and high-spatial resolution X-ray elemental mapping on a FEI Quanta 450 field emission gun SEM, equipped with a Bruker QUANTAX 400 XFlash detector. Later, at the Department of Geosciences, University of Padua we collected electron backscattered diffraction patterns (EBSD) from the alloy phases on a CamScan 2500 SEM using a conventional LaB_6_ source and equipped with a NordlysNano EBSD detector (Oxford Instruments) and coupled with Channel 5.12 EBSD software. The sample was further polished using a NaOH colloidal silica suspension (0.06 µm) for ~10 hours to remove surface arising from mechanical polishing. Operation conditions were 25 mm working distance, 15 kV beam acceleration and 10 nA probe current. The resulting EBSD-patterns contained up to 10 Kikuchi bands. A minimum number of 6 bands used to verify identification accuracy. Finally, the particle was sent to The Natural History Museum (NHM), London for high precision wavelength dispersive spectrometry (WDS) electron microprobe analyses (EMPA), recording major and minor element compositions from the alloy and silicate phases. These data were collected using a JEOL JXA-8530F field emission gun electron microprobe, operating at 10 kV and probe currents of 10 nA. Detection limits for Al, Na and Mg are typically <100 ppm, equivalent to 0.01 wt%, while Si, Cr, Mn, Ca, S and Ti have limits on the order of 200–400 ppm (0.02–0.04 wt%). Higher detection limits are seen for Cu, Ni and Co at 700–1200 ppm (0.07–0.12 wt%). In some cases, multiple analyses can be averaged to derive a composition which includes quantitative detection of trace elements below the detection limit of the analytical equipment, but which remains meaningful.

## Results

**Figure 2:**
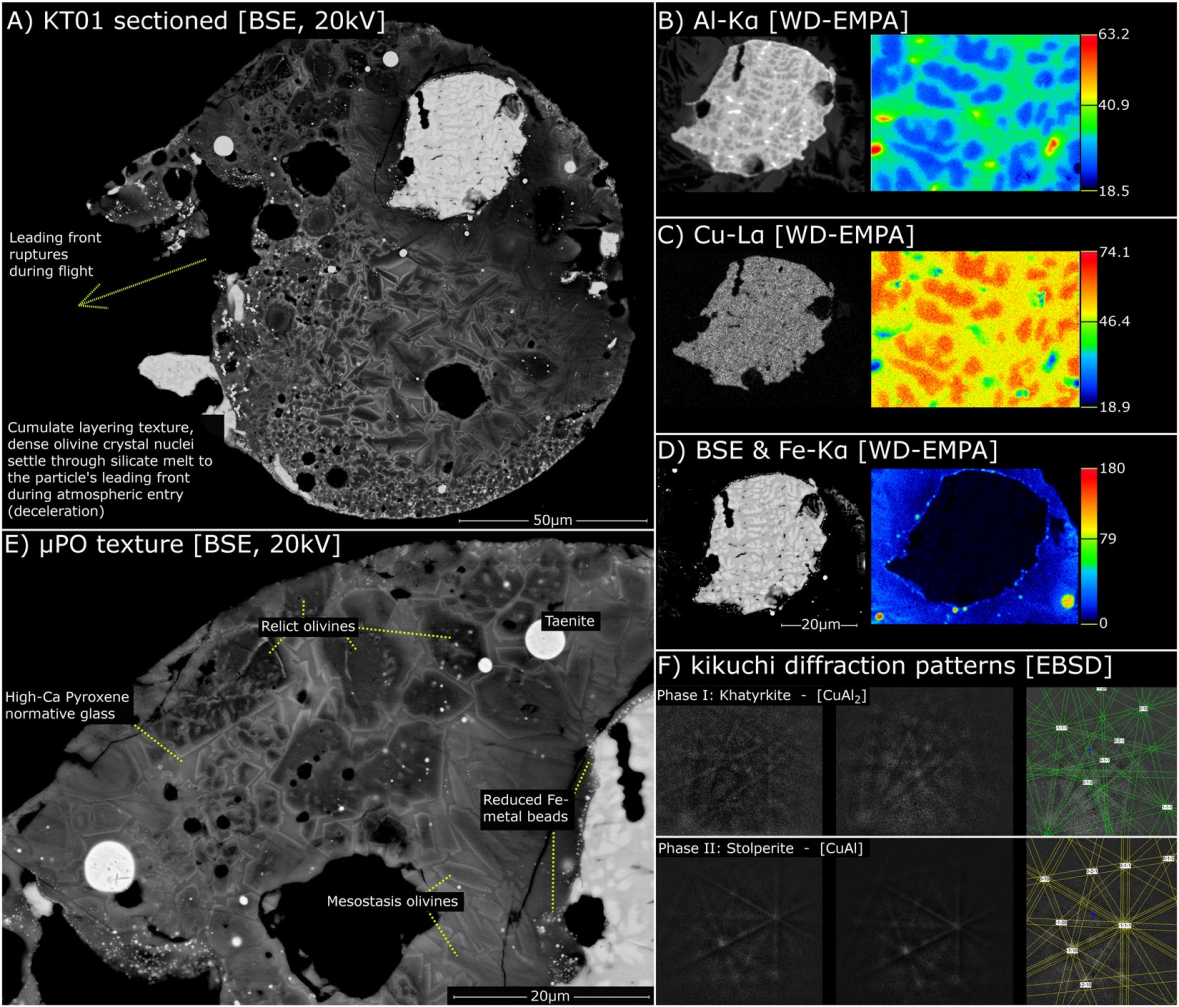
Annotated BSE images and WD-EMPA single element (false colour) maps. (**A**) whole particle cross-section illustrating the cumulate texture and site of in-flight rupture. (**B**–**D**) single element Al, Cu and Fe maps (false colour numerical scales displayed in counts per pixel). (**E**) Magnified particle texture, illustrating the main phases. (**F**) Kikuchi diffraction patterns from the alloy phase.

Particle KT01 (Fig. 2) is a silicate-dominated S-type cosmic spherule with a micro-porphyritic (μPO) texture under the classification system of Genge *et al*.^1^. The particle has an oblate spheroid shape (Fig. 1D) with external dimensions of 140 μm by 90 μm and a broadly circular exposed cross-section with a diameter of ~130 μm. Inside, this particle has a *cumulate texture* characterised by a dense clustering of phenocrysts concentrated within a single hemisphere of the cosmic spherule. Often texture this co-occurs with a higher abundance of vesicles in the corresponding hemisphere. In KT01 the cumulate texture is discernible but not well-developed.

The bulk composition for KT01 is chondritic for major element abundances (Table 1 A14, Fig. S1), while the mineralogy is composed of normally zoned euhedral dusty olivine (micro)-phenocrysts (<10 μm) interspersed with less abundant equant magnetite crystallites. These phases are suspended within a Ca-rich (~5.80 wt% ± 0.6 wt%[1σ]) and Na-bearing (up to 0.76 wt%) silicate glass with a pyroxene composition (Table 1: A8, augite: En9, Fs68, Wo23). Mesostasis (neo-formed) olivine crystals are Mg-rich (Fo64–77) and have Ca (0.18–0.76 wt%), Cr (0.21–0.38 wt%), Mn (0.07–0.27 wt%) and Ni (0.06–0.39 wt%) minor element contents and notably high Al (0.21–1.43 wt%) and Cu (<0.59 wt%) concentrations (Table 1: A7). Meanwhile magnetite grains contain Al and Ni as evidenced from their EDS-maps (Fig. S2). All these phases are mesostasis products, formed by quench cooling during atmospheric entry^1^.

**Table 1:** Geochemical data from all phases within this micrometeorite (either *standardless EDS [normalized to 100* *wt%]* or WD-EMPA).

ID	Phase	*Instr*.	*Analy. type*	*Wt%*															*At%*	
				Na	Mg	Al	Si	S	Ca	Ti	Cr	Mn	Fe	Co	Ni	Cu	O	Total	Stoichiometry	Ratios
A1	Relict silicates	EMPA	Point [1]	0.01	28.66	0.04	18.87	—	0.11	—	0.05	0.09	8.40	—	—	0.50	42.99	99.73	(Mg,Fe)2(Si)O_2_	Fo88.7
A2	Relict silicates	EMPA	Point [1]	—	27.66	0.06	18.96	0.01	0.06	—	0.16	0.19	9.68	—	0.22	—	42.94	99.95	(Mg,Fe)2(Si)O_2_	Fo86.8
A3	Relict silicates	EMPA	Point [1]	0.01	26.77	—	17.81	—	0.12	0.04	0.02	0.21	11.38	—	—	—	41.34	97.72	(Mg,Fe)2(Si)O_2_	Fo84.8
A4	Relict silicates	EMPA	Point [1]	0.02	25.77	0.18	18.97	—	0.13	—	0.15	0.16	10.99	0.22	0.08	—	42.21	98.88	(Mg,Fe)2(Si)O_2_	Fo84.3
A5	Relict silicates	EMPA	Point [1]	0.01	25.80	0.03	18.84	—	0.15	—	0.03	0.12	12.94	0.09	0.12	0.14	42.38	100.65	(Mg,Fe)2(Si)O_2_	Fo82.1
A6	Relict silicates	EMPA	AVG [12]	0.01	26.66	0.08	18.51	—	0.12	0.01	0.08	0.14	11.09	0.08	0.11	0.18	42.14	99.22	(Mg,Fe)2(Si)O_2_	Fo84.6 ± 3.7 [σ1]
A7	Neoformed silic.	EMPA	AVG [7]	0.02	21.75	0.52	17.06	0.01	0.36	0.02	0.27	0.16	18.35	0.07	0.22	0.17	40.04	99.02	(Mg,Fe)_2_(Si,Al)O_2_	Fo73.1 ± 3.1 [σ1]
A8	Glass [Hg.-Ca Px]	EMPA	AVG [8]	0.64	1.32	8.66	16.35	0.09	5.77	0.25	0.20	0.24	23.88	0.07	0.12	0.36	37.22	95.17	(Ca,Fe)(Si,Al)_2_O_6_	En8.6, Fs68.2, Wo23.0 ± 5.8, 5.6, 1.6 [σ1]
A9	Fe-metal	EDS	Point [1]	—	—	—	*0.1*	—	—	—	—	—	*98.8*	—	*0.2*	*0.5*	*0.3*	*100.0*	Fe	Fe/Ni = 553
A10	Taenite	EDS	Point [1]	—	—	—	—	—	—	—	—	—	*47.4*	—	*49.9*	*2.5*	*0.2*	*100.0*	FeNi	Fe/Ni = 1.0
A11	Khatyrkite	EMPA	AVG [4]	—	—	43.37	0.14	—	—	—	—	—	1.01	—	0.09	54.32	—	98.94	CuAl_2_	Al/Cu = 2
A12	Stolperite	EMPA	AVG [6]	—	—	36.01	0.05	—	—	—	—	—	1.44	—	0.19	62.18	—	99.87	CuAl - Cu_2_Al_3_	Al/Cu = 1.4
A13	Alloy	EMPA	Bulk [10]	—	—	42.04	0.12	—	—	—	—	—	1.08	—	0.11	55.75	—	99.11	Cu_5_Al_9_	Al/Cu = 1.8
A14	Silicate bulk	EDS	Bulk [1]	—	*15.3*	*11.1*	*17.0*	—	*1.8*	—	—	—	*20.7*	—	*0.5*	*8.4*	*24.8*	*100.0*	—	—

We use the “—” symbol to denote elements below the detection limits. Square brackets indicate the number of analyses averaged to generate the given composition.

**Figure 3:**
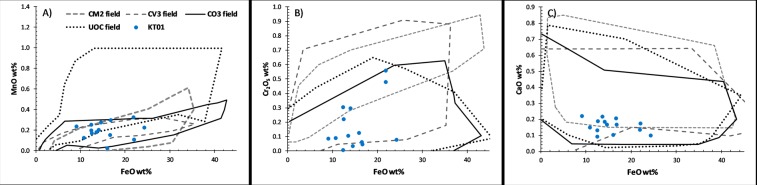
Minor element data from relict anhedral dusty olivines (point analyses from the grain cores) and comparison with carbonaceous/ordinary chondrite meteorites. These minor element compositions are best fit to the CO3 field, with all datapoints falling within their range. UOCs and CV3 chondrites are also possible matches but less likely, while the CM2 range is inconsistent with the observed compositions.

In contrast, KT01 retains some larger relict (unmelted) olivine phenocrysts (Table 1: A1**–**6, Fig. 3) with anhedral irregular morphologies and broadly circular shapes (Fig. 2E). These are recognisable from their lower back-scatter potential, reflecting the Mg-rich cores (Fo76–90). They also have distinctive minor element composition, composed of Ca (<0.06–0.16 wt%), Mn (0.02–0.23 wt%) Cr (<0.29 wt%) and Ni (<0.35 wt%) with minimal Al (<0.18 wt%) and Cu (<0.5 wt% but typically below the analytical detection limit [<0.12 wt%]) (Fig. 3). In addition, these relict grains have “*dusty olivine*” textures, identified by the presence of poikilitic submicron rounded Fe-metal droplets within their cores.

Aside from the silicate components, accessory metallic and intermetallic phases are present. This includes two round droplets of Fe-Ni metal with taenite compositions (Fig. 1E, Table 1: A10, Fe 47.4 wt%, Ni 49.9 wt% and Cu 2.5 wt%) and diameters between 5–10 μm. There are also abundant dispersed metal droplets with homogenous textures and bright backscatter potential - these are almost pure Fe droplets which range in size from the nanoscale up to ~5 μm diameter and contain less than 0.5 wt% Ni. Both Fe-bearing phases are unusual compared to the Fe-Ni metal grains commonly found in cosmic spherules which typically have kamacite compositions, while taenite and pure Fe are rare^1^.

Finally, the phases which make this particle unique are a series of large irregular-shaped Al-Cu-Fe alloys, which form three distinct masses. The largest mass (Fig. 2A–D and Fig. S3A) has a rounded margin along part of the alloy complex but a broken with irregular shape along the remaining perimeter. This mass reaches ~44 × 34 μm in size. The next largest mass lies at the particle margin (Fig. S3B) and has a rounded margin and elongated “*tail*” which runs along the spherule’s outer surface decreasing in thickness and forming a tapering extension. Finally, the smallest mass (10 × 3 μm, Fig. S3C) is elongated with indistinct margins. These Al-Cu-Fe alloys are composed of two closely related phases that have co-crystalized with an equiaxed dendritic and cloverleaf morphology^17^, they are easily resolved by their distinct difference backscatter potentials. The two phases are identified by atomic stoichiometry as khatyrkite ([Cu,Fe]Al_2_) (the darker phase, Table 1: A11, Cu 54.32 ± 1.6 wt% [1σ], Al 43.37 ± 1.5 wt% [1σ], Fe 1.01 ± 0.20 wt% [1σ], here appearing as CuAl_2_ – and without detectable Zn) and stolperite, CuAl (the brighter phase, Table 1: A12, Cu 62.18 ± 2.2 wt% [1σ], Al 36.01 ± 1.8 wt% [1σ], Fe 1.44 ± 0.1 wt% [1σ]). Minor element data reveal (atomic) Fe/Ni ratios of 11.8 and 8.0 for the khatyrkite and stolperite minerals respectively. The identities of these two alloy phases were further confirmed using EBSD pattern recognition (Fig. 2F), which also demonstrated that the two phases have, in all instances, the same crystallographic orientation.

In the largest mass, (Fig. 1A–D) the two phases co-occur with an approximate ratio of 80:20 (by exposed surface area), with khatyrkite as the major phase and stolperite as the minor phase. Using the averaged compositions for each phase this suggests the Al-Cu-Fe alloy’s bulk composition is approximately: (Cu 55.75 wt%, Al 42.04 wt% and Fe 1.08 wt%, Table 1: A13).

## Discussion

### Al-Cu-Fe alloys and the Khatyrka meteorite

The most unusual feature of the KT01 micrometeorite is the presence of exotic Al-Cu-Fe intermetallic alloys. These phases are exceptionally rare in nature, having been previously described only from a single terrestrial outcrop – The Koryak Mountains, Russia^15^. At this site intermetallic alloys were found to be weathering out of a single disintegrated meteorite fall: the Khatyrka meteorite.

The Khatyrka meteorite is a member of the CV3-oxidised subgroup of (anhydrous) carbonaceous chondrites and is composed of fayalite (Fo40–60) Ca, Fe-pyroxenes, andradite, nepheline, sodalite, Fe,Ni-sulfides, Ni-bearing magnetite and Ni-rich metal. This meteorite is a disintegrated find, discovered in 2011 from Eastern Siberia, Russia as a series of detrital grains found among clays in the Koryak Mountains^15^. In total only 10 grains of the Khatyrka meteorite are known and all are approximately millimetre-sized, however, they include fragments of matrix, type IA Mg-rich, reduced and metal-bearing chondrules as well as refractory CAIs and, notably embedded and cross-cut^18,19^ exotic micron-sized grains of Al-Cu-Fe alloys.

The Khatyrka meteorite was recovered during a dedicated research mission to the Koryak Mountains^20^ in search of the mineral Khatyrkite and other related phases that had previously been discovered there (and only there) in 1985 by placer deposit platinum miners^21^. Thus, it is the presence of these unusual metallic alloys, which are not known to occur elsewhere in nature including in any other meteorite, that make Khatyrka unique. The family of Al-Cu-Fe alloys occur as accessory phases in Khatyrka and include: khatyrkite, stolperite (CuAl) and its polymorph cupalite as the most abundant alloys^22^; in addition more complex phases include: kryachkoite ([Al,Cu]_6_[Fe,Cu]), hollisterite (Al_3_Cu)^23^, icosahedrite (Al_63_Cu_24_Fe_13_)^24^, decagonite (Al_71_Ni_24_Fe_5_)^25^, proxidecagonite (Al_34_Ni_9_Fe_2_)^26^, and steinhardtite (Al_38_Ni_32_Fe_30_)^27^.

Despite their rarity, the natural Al-Cu-Fe alloy system has received significant attention since its discovery owing to both their unusual geochemistry and crystallography. First, the co-occurrence of Cu and Al together is highly unusual in either a terrestrial or cosmochemical setting. This is because Cu is a moderately volatile (1030 K)^28^ chalcophile element while Al is a highly refractory (1677K)^28^ lithophile element, thus their different geochemical behaviours make them highly unlikely to partition into the same minerals. For example, in chondrites formed by condensation and repeated heating cycles in the protoplanetary disk, Al partitions into refractory anorthite and spinels while Cu co-occurs with sulphides as a late-stage volatile addition. Furthermore, both elements have low oxidation potentials meaning they are easily converted into cations.

The occurrence of native Al on the Earth’s surface is thus rare owing to the extremely reducing conditions required for its formation^22^. Despite this, currently 28 occurrences are reported from the Earth (and Moon system) as evidenced by the Mindat website^29^. These are primarily products of post volcanic or magmatic hydrothermal activity resulting in economic ore mineralisation. Likewise, reports of Al-bearing alloys in cosmochemical settings are rare but include the Zhamanshin astrobleme crater on Earth^30^, the shocked Suizhou L6 chondrite^31^ and in the carbonaceous, diamond-bearing stone “*Hypatia*”^32^.

Finally, two of the alloy phases discovered in the Khatyrka meteorite (icosahedrite and decagonite) are quasicrystals; a recently discovered new group of solid matter with atomic structures that are ordered but not periodic, meaning they exhibit short-range translational order combined with high order rotational symmetries that are otherwise forbidden for crystalline materials – namely fivefold symmetry in two-dimensions and icosahedral symmetry in three-dimensions^24,25^. We did not observe quasicrystalline phases within KT01, likely due to the low amounts of Fe in the alloy insufficient to stabilize quasicrystals - despite their absence the petrographic similarities between the two intermetallic alloy-bearing extraterrestrial samples are striking, both presenting with exactly the same major minerals (khatyrkite and stolperite) and dendritic quench textures and found among a carbonaceous chondrite host – they may therefore originate from the same parent body.

### The parent body source of KT01 micrometeorite, evidence from particle texture and relict silicates

KT01 exhibits a μPO texture, which is associated with carbonaceous chondrite source bodies^33^. Likewise, data from the relict silicate cores confirms an anhydrous chondrite source.

Minor element compositions from anhydrous silicates are often distinct and may be used to relate individual micrometeorites to specific parent body classes. Here we compare the MnO, Cr_2_O_3_ and CaO contents of the relict silicates in KT01 against the compositional ranges for CM2, CV3, CO3 chondrites and unequilibriated ordinary chondrites (UOCs) (Fig. 3). This comparison reveals a best match to the CO3 chondrite population. While most individual analyses also lie within the compositional range of other chondrite groups, all data points across all three plots are best explained by a CO3 precursor, making this parent body the most likely group.

The relict silicates in KT01 are dusty olivines, formed by the solid-state exsolution of Fe under reducing conditions through heating at subsolidus temperatures^34^ they are closely associated with type I chondrules^35^ and document multi-generation heating and redox cycles within the chondrule reservoir^34^. Dusty olivines are commonly associated with ordinary chondrites but are also known from CV chondrites^36^ and CO chondrites where they are rare components, found only within chondrule cores^37^.

The low Cu and Al contents of the relict silicates, combined with a lack of correlation between Cu and Al abundance demonstrates that the relict silicates formed in isolation from the Al-Cu-Fe alloy and thus, most likely prior to its introduction into the mineral assemblage. Furthermore, we can rule out the formation of the dusty core textures within these silicates as a product of rapid reduction during atmospheric entry because, in all instances, the dusty olivines within KT01 are surrounded by thin homogenous Mg-rich rims several microns thick. These Mg-rich rims are further held within the Fe-enriched neo-formed rims that grew during atmospheric entry. Thus, if the dusty olivines had been generated during atmospheric entry we would expect the reduced Fe-droplet-bearing cores to be in direct contact with the Fe-rich overgrowth rims, however, because this is not the case, the dusty olivines must be a parent body feature. To further support this argument, we note that previous experimental data attempting to recreate dusty olivines were only successful after the Fe-bearing olivines had been heated, close to their sub-solidus temperatures, for timescales between several minutes and several hours^38^. Such heating scenarios are inconsistent with the heating regimes present during atmospheric entry which last for <10 seconds^39^.

### The atmospheric entry of KT01

Cosmic spherules experienced a high degree of melting and recrystallization during atmospheric entry^1^. Whilst in a molten state, they form spherical droplets, pulled in by surface tension. Meanwhile, their internal components separate as a result of immiscibility^40^ and density contrast^41^. Under reducing conditions, arising from the thermal decomposition of carbonaceous phases (Fe-Ni) metal beads commonly form, coalesce and can potentially escape by exit through the leading front of the particle^40,42^. This process is promoted by high apparent deceleration which acts to magnify density contrasts. In KT01 we identified a weak cumulate layering texture (Fig. 2A), this is evidence of orientated flight during atmospheric entry and demonstrates that the denser phases (olivine, metal and [fragments of the] intermetallic alloys) within the spherule migrated under gravitational force to the leading front of the particle. Furthermore, cumulate textures potentially indicate relatively high entry velocities (>16 kms^−1^), typical of highly eccentric orbits of dust released from the main and outer asteroid belt^43^. Thus, KT01 may have originated from either an asteroid in the middle or outer zone of the main belt.

In contrast to Fe-Ni metal bead formation, the Al-Cu-Fe alloys are unlikely to have been formed by *in-situ* reduction during atmospheric entry, as this would require extremely low oxygen fugacities to form such beads^22^ – close to the Si-SiO_2_ buffer. Such conditions are highly unlikely and could inhibit the crystallization of observed mesostasis olivine. In addition, we know of no suitable donor phases within a chondritic precursor that could thermally decompose to provide the required quantities of Cu and Al. Instead, the intermetallic alloys most likely existed, prior to atmospheric entry, as an original component of the parent body assemblage and as a single mass.

The Al-Cu-Fe alloy system has a liquidus temperatures of ~1100 °C^44^ this is far below the chondritic solidus temperature (~1350~1700 °C)^45^. Thus, the alloy would melt early in the particle’s flight (and subsequently cool late). Upon melting, the alloy would form a droplet - generating the observed rounded perimeter. In KT01, the alloy later broke apart into several smaller pieces, possibly caused by thermal stresses during cooling^46^, this resulted in the irregular surface along the left-hand side of the largest mass – annotated in Fig. 1A as well as the generation of smaller fragments which then travelled downwards, towards the leading edge of the spherule.

A single fragment of this alloy was ultimately exposed at the spherule margin, adjacent to where the spherule later ruptured (Fig. 1A). This mass appears to have subsequently flowed out and upwards following the spherule’s external perimeter, forced by atmospheric drag. This behaviour requires the alloy to have a lower viscosity than the silicate melt. Near-identical behaviour, in which a sulfur-bearing Fe-Ni bead flows out of the particle’s leading edge and up along the particle perimeter of S-type cosmic spherules are relatively common (Fig. S4) and first reported by Taylor *et al*.^42^.

Simultaneously with fragmentation, the alloy mass would also have experienced melting, redox reactions and geochemical interaction with the other phases in the micrometeorite. Evidence of geochemical exchange is clearly seen at the contact of the Al-Cu-Fe alloys and the surrounding matrix where a continuous series of small (<1 μm) rounded Fe droplets (that lack Ni) occur (Fig. 1A,C,E). These droplets attest to an *in situ* redox reaction between the silicate melt and Al-Cu-Fe intermetallic alloys. They were therefore generated by the reduction of Fe^2+^ ions, initially held within the silicate melt, forming neutrally charged metallic Fe^0^ atoms which were then immiscible in the silicate melt. This reduction reaction was facilitated by the extremely low-fugacity conditions supported by the presence of metallic Al (Fig. 5 in Bindi *et al*.)^22^.

We deem this scenario more likely than a situation in which the pure Fe-metal droplets are primary feature of the pre-atmospheric parent body as found within some chondrules. This is because the Fe droplet sizes are smaller significantly smaller than those typically found in chondrules^47^ and because the droplets occur in notably high abundance adjacent to the reducing alloy than throughout the remainder of the particle.

In addition to redox reactions, wholesale melting and dissolution of alloy into the silicate melt has also occurred, as determined by the high abundance of (Al-bearing) magnetite spinels (Fig. S2), and the strikingly high concentrations of Al that have partitioned into the mesostasis olivines (up to 1.43 wt%, Table 1: A7). Furthermore, the element maps (Fig. 1B–D, Fig. S2) show leaching of Al from the alloys into the surrounding silicate melt (now a glass), with a relative retention of Cu (identified by sharper boundaries and clear hotspot zones in the element maps). This is most clearly seen in the supplementary file whole-particle EDS Al-Cu map where a diffuse zone of Al-enrichment within the silicate glass can be identified, this may be expected given the compatibility of Al, a lithophile element within a silicate melt, and ultimately acts to unmix the Al-Cu-Fe alloy. This behaviour demonstrates that Al-Cu-Fe alloys are unstable in molten chondritic systems and would unmix if the two melt phases were left to equilibrate over even short timescales. Thus, the initial formation mechanism which generated the intermetallic alloy either occurred in isolation (in a closed system separate from the chondritic components) or under extreme environmental conditions and rapid crystallization.

**Figure 4:**
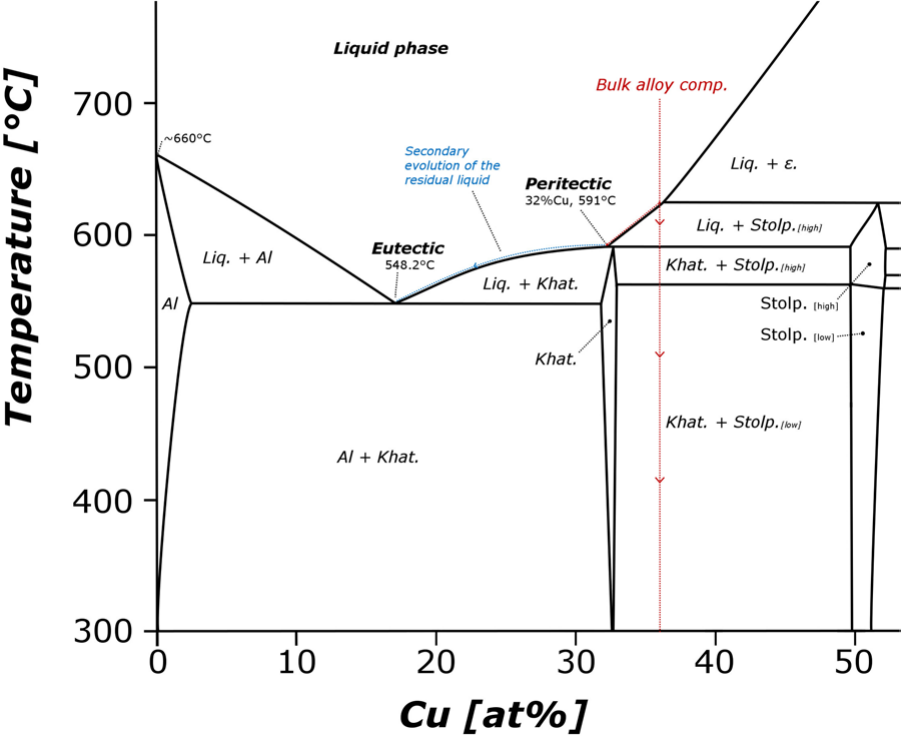
Cu-Al system binary phase diagram reproduced after significant alteration from Murray (1985)^40^, showing a restricted section of the diagram, relevant to Al-rich compositions. The cooling history of the bulk Al-Cu-Fe alloy during atmospheric entry is marked by the dashed red line and starts at high temperatures, assuming a completely molten state. Cooling at the peritectic generates the observed two-phase intermixed assemblage. Stolperite forms first and subsequently reacts with the remaining melt to form khatyrkite, leading to both minerals displaying the same crystallographic orientation and smooth rounded margins to the (stolperite) dendrites. However, since some of stolperite grains become encased in a shell of khatyrkite, they are unable to react under equilibrium at the peritectic resulting in Al-enrichment of the residual liquid, whose composition then evolves down towards the eurtectic (dashed blue line) and crystalizes khatyrkite plus (pure) Al. This secondary non-equilibrium cooling is necessary to explain the observed three phase composition of the alloy bead seen in KT01. (**Note**: for the Al-Cu-Fe system Fe concentrations below 10 wt% have little effect on the cooling behaviour allowing us to assume a 2-element system). Additional complexity relevant at higher Cu contents (>50 at%) has been omitted for simplicity.

Late in the micrometeorite’s atmospheric entry flight and low in the atmosphere (<70 km) peak temperatures rapidly decrease^39^, resulting in cooling and crystallization. We previously noted that EBSD revealed the two Cu-Al phases have the same crystallographic orientation, indicating they grew by a peritectic reaction under near-equilibrium conditions (Fig. 3 in Hollister *et al*., 2014)^18^. By calculating the modal abundances of the two phases within the largest alloy bead (Fig. 1A-EE) and using the average phase compositions for khatyrkite and stolperite we calculated the bulk composition of the alloy system in KT01 (Table 1: A13). This revealed a bulk (At% Cu/Al ratio of 0.56, Cu36%: Al64%) stoichiometrically close to the peritectic point of the binary Cu-Al system at ~550 °C (Cu30%: Al70%, Fig. 4 in Murray, 1985^44^) and requires that the Cu-Al alloy bead cooled late in the flight, at relatively low temperatures and as one of the last phases within the micrometeorite – consistent with the disparate solidus-liquidus temperatures of chondritic melts (1350–1900 °C)^45^ and the Al-Cu system (550–1100 °C)^44^. Cooling at the peritectic reaction would first crystalizes stolperite, before reacting with the remaining melt to form interstitial khatyrkite (Fig. 4, red dashed line). However, because we also observe small regions of pure Al (Fig. 2B) in the X-ray maps this requires some degree of non-equilibrium cooling. Due to the rapid kinetics of the reaction, some stolperite was unable to react to completion, failing consuming all the liquid phase while at the peritectic point before the bead cooled further. This metastable preservation is possible if stolperite grains become encased and protected by a shell of overgrown khatyrkite, at which point rapid cooling prevents the system from achieving equilibrium before the temperature decreases beyond the peritectic. The resulting residual liquid is Al-rich, and its composition would then continue to evolve down to the eutectic, thereby crystalizing isolated regions of pure Al (dashed blue line). This cooling scenario allows for the production of all three phases (khatyrkite, stolperite and pure metallic Al) coexisting in a single alloy bead.

## Implications

The absence of intermetallic Al-Cu-Fe alloys in any other anhydrous carbonaceous chondrite or indeed any meteorite, among a population over 35,000 certified members strongly implies that these intermetallic alloys are not native components of the chondritic assemblage. Likewise, evidence suggests that the Al-Cu-Fe alloy (and associated quasicrystals) in Khatyrka formed after the main components of CV chondrite. Evidence for their later formation age is three-fold, firstly the radiometric (U-Th-Pb dating) ages of olivine crystals within the Khatyrka meteorite record a resetting event <600 Ma ago^48^. This requires a significant impact event on the parent asteroid. Secondly, experimental tests demonstrate that quasicrystals with compositions similar to those reported in the Khatyrka meteorite (e.g. Al_68–73_Fe_11–16_Cu_10–12_Cr_1–4_Ni_1–2_) can be generated and remain stable under high temperature and pressure (>14 GPa and 1400 °C)^49^ typical of impact conditions. Finally, Khatyrka contains diagnostic index minerals associated with high shock pressures. These include ahrensite and stishovite^18,50^ and require minimum pressures of >10 GPa and temperatures >1200 °C.

The Al-Cu-Fe alloys reported in Khatyrka and the KT01 micrometeorite are therefore exotic non-chondritic components which formed after the initial host’s accretion and were delivered most probably by an impact event. These exotic intermetallic alloys may originate as extrasolar objects, sourced from outside our own solar system. Such a radial suggestion would, however, require significant additional isotopic data – showing extreme excursions far beyond the typical values observed for native solar system objects before we could confidently support such a hypothesis. Future experiments, analysing the isotopic ratios of Al, Cu and O in the KT01 particle are therefore planned to investigate this possibility.

As only the second independent discovery of these phases, our micrometeorite (KT01) demonstrates that such alloy phases are not a single occurrence but may be more common than previously thought as a rare but distinct petrographic phase within chondritic bodies, which could potentially record an influx of interstellar material to the inner solar system. If correct, this would demonstrate that chondrites not only record the early solar system evolution but also act as geological repositories for captured material from other star systems.

## Conclusions

We provide a petrographic description of a unique micrometeorite (KT01), which contains an exotic two-phase Al-Cu-Fe alloy composed of the highly unusual minerals: khatyrkite and stolperite. They show clear evidence of quench-cooled growth from a geochemically isolated melt at relatively low temperatures (~550 °C, the peritectic). The host micrometeorite is an S-type relict-bearing μPO cosmic spherule with a cumulate texture. Relict anhydrous silicate crystals inside KT01 are dusty olivines with minor element contents suggestive of a CO parent body source. The absence of appreciable Cu or Al within the relict crystals demonstrates that these grains grew from a typical chondritic igneous melt and predate the Al-Cu-Fe alloy.

This study represents only the second independent discovery of exotic Al-Cu-Fe alloys within a chondritic setting, thereby supporting the original discovery from the Khatyrka (CV3) chondrite. Our study demonstrates that such alloys are present on at least two chondritic parent bodies (i.e. the CV [Khatyrka] and CO [KT01] parents). Their origin could be interstellar although further study is required. If so, they would reflect the delivery of extrasolar material to the inner solar system and its preservation (in an impact-altered state) on various asteroidal bodies.

## Supplementary information

Supplementary materials
