# Supplementary material for: A unique CO-like micrometeorite hosting an exotic Al-Cu-Fe-bearing assemblage – close affinities with the Khatyrka meteorite
Source: Sci Rep. 2019 Aug 27;9:12426. doi: 10.1038/s41598-019-48937-0 (PMC6711995; doi:10.1038/s41598-019-48937-0)

Supplementary materials: *A unique CO-like micrometeorite hosting an exotic Al-Cu-Fe-bearing assemblage – close affinities with the Khatyrka meteorite*

Suttle, M.D.[1](*corresponding author*), Twegar, K.[2], Nava, J.[3], Spiess R.[3], Spratt, J.[4], Campanale, F.[1,5] , Folco, L.[1] – martindavid.suttle@dst.unipi.it

---

[1.]Dipartimento di Scienze della Terra, Università di Pisa, 56126 Pisa, Italy
[2.] Department of Chemistry, Istanbul Technological University, 34467 Istanbul, Turkey
[3.] Dipartimento di Geoscienze Via Gradenigo, 6 - 35131 Padova
[4.]Department of Earth Science, The Natural History Museum, Cromwell Rd, South Kensington, London SW7 5BD, UK
[5.] Center for Nanotechnology Innovation@NEST, Istituto Italiano di Tecnologia (IIT), Piazza San Silvestro 12, 56127 Pisa, Italy

---

Figures

Fig.S1. Spider diagram of KT01 – inferred particle bulk composition (by EDS, Table.1: A14) and mesostasis silicate glass phase (by WD-EMPA, Table.1: A8).

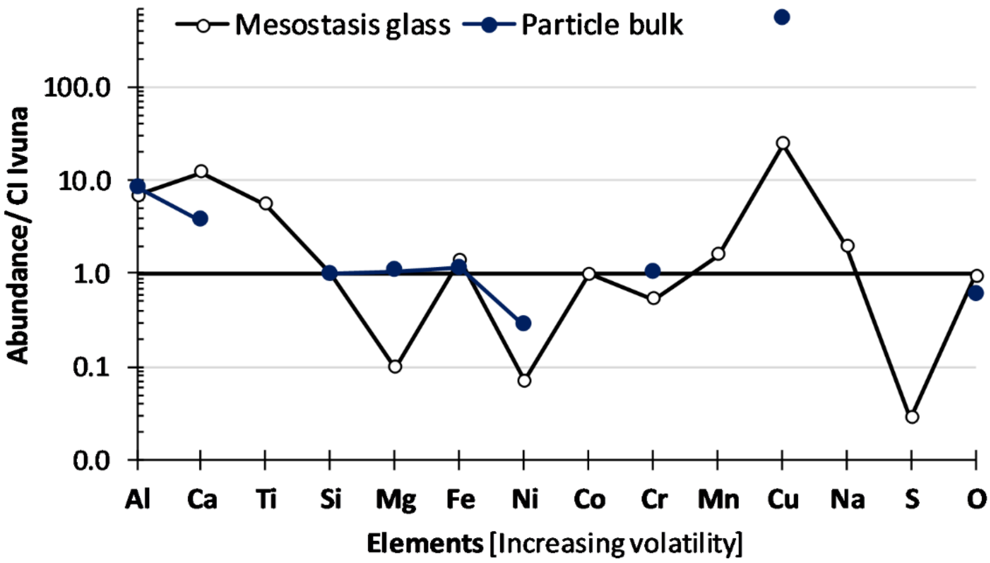

Fig.S2. Whole particle combined element EDS maps showing the distribution of major elements (Mg-Si-Fe [B, C]) as well as Cu and Al (A). The dual element Cu-Al map demonstrates the unmixing (primarily of Al) from the alloy during atmospheric entry flash heating.

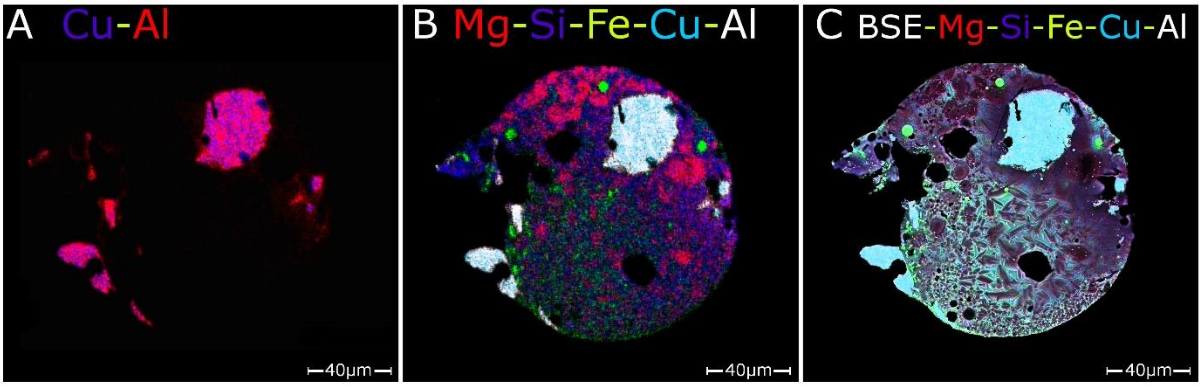

Fig.S3. Back-scatter electron images of Al-Cu-Fe alloy beads present within KT01. The three beads vary in size, shape and edge properties. The largest bead and bead at the perimeter both clearly show a 2-phase dendritic crystallization texture while the smallest bead has indistinct margins and a faint outlines of internal dendrites, this bead appears to be dissolving into the surrounding silicate matrix.

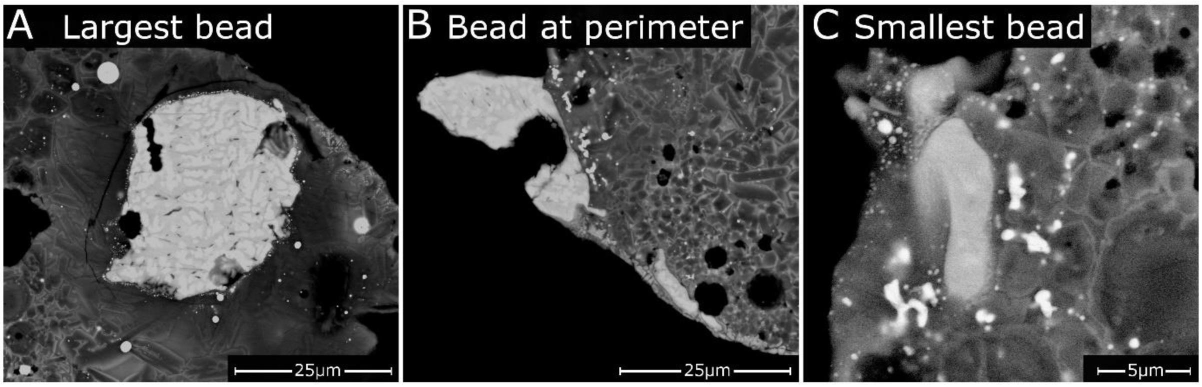

Fig.S4. Metal bead and metal-sulfide bead separation and smearing events observed in S-type cosmic spherules (From Larkman Nunatak and the TAM collections). Owing to immiscibility, Fe-Ni and siderophile elements geochemically segregate from the silicate melt. This phase is denser than the silicate and thus migrates to the leading edge of the micrometeorite “pulled” downwards by gravitational force. The bead also has a lower viscosity than the silicate melt allowing it to flow out of the particle where drag forces then smear the bead out and “upwards” along the particle’s external surface. This process has been described previously for S-type micrometeorites by Genge and Grady (1998)[36] and Taylor et al., (2011)[38].

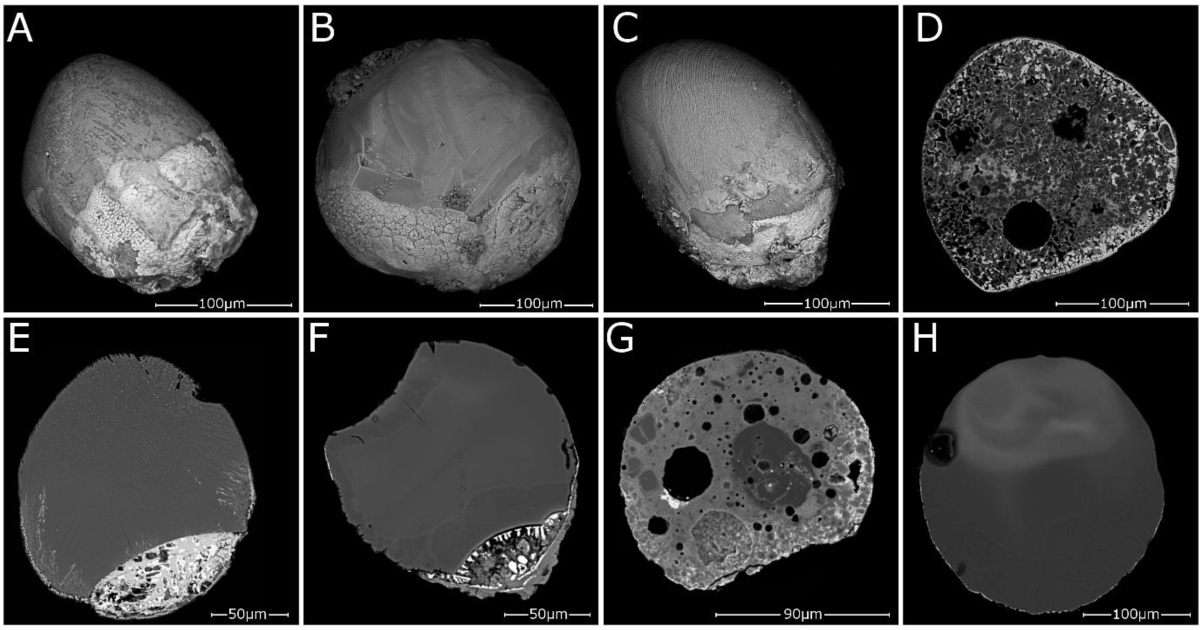

Supplement: Supplementary file 1 — Supplementary materials [file 41598_2019_48937_MOESM1_ESM.pdf]
